# What increase in modern contraceptive use is needed in FP2020 countries to reach 75% demand satisfied by 2030? An assessment using the Accelerated Transition Method and Family Planning Estimation Model

**DOI:** 10.12688/gatesopenres.13125.1

**Published:** 2020-07-21

**Authors:** Niamh Cahill, Michelle Weinberger, Leontine Alkema

**Affiliations:** 1 Department of Mathematics and Statistics, Maynooth University, Maynooth, Ireland; 2 Avenir Health, Washington D.C., USA; 3 Department of Biostatistics and Epidemiology, University of Massachusetts Amherst, Amherst, USA

**Keywords:** Planning Estimation Model (FPEM), demand satisfied, modern contraceptive use, target setting

## Abstract

**Background:** Sustainable Development Goal 3.7 aims to ensure universal access to sexual and reproductive health services. One suggested benchmark is to have at least 75% of the demand for contraception satisfied with modern methods (DS) in all countries by 2030. The translation of DS-based targets into targets for the modern contraceptive prevalence rate (mCPR) is needed to make targets actionable.

**Methods:** We propose the Accelerated Transition (AT) method for determining the mCPR needed to reach demand-satisfied targets by 2030. The starting point for this method is the projection of DS under “business as usual” using the one-country implementation of the Family Planning Estimation Model (FPEMcountry). For countries in which the DS target is projected to be later than 2030, the AT method assumes that meeting the DS target by 2030 requires an acceleration of the contraceptive use transition such that the DS target, and its associated mCPR, will be reached in 2030 as opposed to the later year. The DS-target-associated mCPR becomes the mCPR target for the year 2030.

**Results: **We apply the AT method to assess progress needed for attaining the 75% DS target for married or in-union women in the world’s poorest countries. For 50 out of 68 countries, we estimate that accelerations are needed, with required mCPR increases ranging from 4.3 to 50.8 percentage points.

**Conclusions:** The AT method quantifies the acceleration needed – as compared to business as usual projections – for a country to meet a family planning target. The method can be used to determine the mCPR needed to reach demand-satisfied targets.

## Introduction

Family planning success is typically measured with two key family planning indicators: the modern contraceptive prevalence rate (mCPR) and unmet need for contraception. However, the combination of these two indicators into the more complete ‘demand satisfied’ indicator can better capture a country’s family planning performance ^1^. Demand satisfied has thus been incorporated into the Sustainable development goals (SDGs) with SDG indicator 3.7.1 ^2^ specifying that the international community should assess the ‘Proportion of women of reproductive age (ages 15–49 years) who have their need for family planning satisfied with modern methods’. One proposed benchmark related to SDG 3.7.1 is to have at least 75% of the demand for family planning satisfied with modern contraceptives in all countries by 2030 ^3^. We refer to this global benchmark of 75% demand satisfied as DS75. Other examples of targets for this indicator allow for country variation and past country experiences to inform country-specific target values ^4^.

“Business as usual” (BAU) projections, referring to projections of family planning indicators based on historical trends, can be used to assess if countries are on target to meet DS75 by 2030. The one-country implementation of the Family Planning Estimation Model (FPEMcountry) ^5– 8^ produces model-based projections of family planning based on parametrization of trends in contraceptive use and unmet need. In summary, FPEMcountry models contraceptive prevalence using logistic growth curves. Estimates of unmet need are obtained by capturing the relationship between contraceptive prevalence and unmet need. BAU projections for DS are obtained from projected contraceptive use and unmet need.

Countries that are not currently projected to meet DS75 by 2030 will require a larger-than-BUA increase in the number of users of modern methods of contraception between now and 2030. Estimating the required increase in mCPR and hence required number of additional users for each country can facilitate planning for the appropriate allocation of resources at the country level. A previous study for states in India has estimated the required increases in mCPR and additional users needed to achieve DS targets using 2030 projections of total demand (contraceptive prevalence plus unmet need) for contraception ^8^. This method combined accelerated growth in modern use with business-as-usual growth in total demand; the method did not account for faster-than-BAU increases in mCPR to coincide with faster-than-BAU increases in demand that are likely to occur due to, for example, increased efforts in family planning programs ^9^. Methods that account for faster-than-BAU increases in demand to coincide with faster-than-BAU increases in mCPR are needed to avoid underestimation of mCPR targets, and thus, failure to meet DS targets.

We present a new method for assessing country-specific levels of mCPR needed to achieve DS targets by 2030, referred to as the accelerated transition (AT) method. The starting point for this method is the projection of DS under BAU using FPEMcountry ^5– 8^. For countries in which the DS target is projected to be later than 2030, the AT method assumes that meeting the DS target by 2030 requires an acceleration of the contraceptive use transition such that the DS target, and its associated mCPR, will be reached in 2030 as opposed to the later year. The DS target-associated mCPR is the mCPR target for the year 2030. Based on mCPR targets, we estimate progress needed for attaining DS targets in terms of a mCPR gap. Using the AT method we assess progress for the countries in the FP2020 initiative ^10^ towards reaching mCPR targets associated with DS75 and quantify the relative acceleration needed to achieve these targets in each country.

## Methods

### Family planning indicators and data

Contraceptive prevalence is measured as the percentage of women who report themselves or their partners as currently using at least one contraceptive method of any type (modern or traditional). Unmet need for family planning is defined as the percentage of women who want to stop or delay childbearing but who are not currently using any method of contraception to prevent pregnancy. Observations of unmet need for family planning in our database are, whenever possible, based on the revised algorithm of the indicator designed to improve comparability within and across countries ^11^. The estimates reported in this study are for women of reproductive age (15–49 years) who were currently married or in a union (referred to as married/in-union women of reproductive age [MWRA]).

Family planning data were obtained from nationally representative household surveys, the Demographic and Health Surveys (DHS), Performance Monitoring and Accountability 2020 surveys, the Multiple Indicator Cluster Surveys, the Reproductive Health Surveys, Contraceptive Prevalence Surveys and World Fertility Surveys ^12^. The estimates presented in this report are based on 558 survey observations of contraceptive prevalence between 1968 and 2019 from 68 countries and 320 survey observations of unmet need for family planning from 66 countries. This survey database was prepared for the FP2020 report ^10^ and stored in the data set contraceptive_use_track20 in FPEMcountry ^7^.

### Model-based projections of Family Planning Indicators using FPEMcountry

The FPEMcountry ^7^ R package implements a one-country version of the Family Planning Estimation Model which combines a Bayesian hierarchical model with country specific time trends to yield estimates of contraceptive prevalence and unmet need for family planning for women aged 15–49 who are married or in a union. The model accounts for differences by data source, sample population, and contraceptive methods included in the measure ^5, 6^. For every country, the FPEMcountry R package models contraceptive prevalence with an expected trend that assumes contraceptive prevalence will begin with a gradual increase, it will subsequently become more rapid and then it will begin to slow down when high levels of prevalence are reached. The parameters that control the trend are estimated hierarchically, such that estimates are based on the data available in the country of interest, and also the sub-regional, regional, and global experience. Distortions are added to capture how rates of change in the observed data (i.e., faster/slower rates of change in contraceptive prevalence) deviate from the rates of change indicated by the expected trend. Projections are informed by recent changes that have occurred in contraceptive prevalence (i.e., the difference between the two most recent surveys) as well as past experience ^6^. Estimates of unmet need are obtained by capturing the relationship between contraceptive prevalence and unmet need. Similar to the model for contraceptive prevalence, a hierarchical approach is used to estimate parameters. Time dependent distortions are added to capture country-specific changes in the level of the indicator ^5, 6^. Estimates and projections of demand satisfied are derived from calculating the ratio of modern contraceptive prevalence to total demand (contraceptive prevalence plus unmet need).

### Accelerated Transition (AT) method

**Figure 1.**
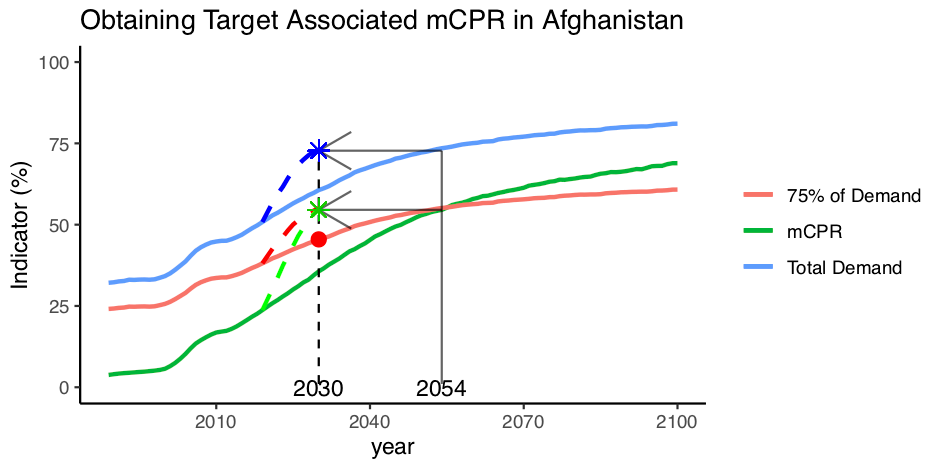
An illustration of the Accelerated Transition and Demand-based methods to obtain modern contraceptive prevalence rate (mCPR) targets for Afghanistan. Business as usual projections (solid lines) and accelerated transition projections (dashed lines) for mCPR, total demand and 75% demand satisfied. Accelerated transition (AT)-based mCPR target and total demand for 2030 are indicated with stars, the demand-based mCPR target (75% of total demand in 2030) is shown in red.

We propose the Accelerated Transition (AT) method for assessing the mCPR level needed to reach a specific DS target. The starting point for this method is the projection of DS under “BAU” using projections, in the case of FP, using FPEMcountry. BAU projections are illustrated for Afghanistan in Figure 1 (solid lines). For countries such as Afghanistan in which the DS target is projected to be later than the target year, the AT method assumes that meeting the DS target by 2030 requires an acceleration of the contraceptive use transition such that the DS target, and its associated mCPR, will be reached in 2030 as opposed to the later year. The DS target-associated mCPR becomes the mCPR target for the year 2030. For example, in Afghanistan, DS75 is achieved in the year 2054, when 75% of demand (red line) overlaps with mCPR (green line) and is equal to 54.6%. Under an accelerated transition (dashed lines), the DS75 target is reached in 2030 instead, and the mCPR is equal to 54.6% in 2030.

### AT measures: relative acceleration, target mCPR, and progress assessments

We let t* be the earliest year in which a country is expected to achieve DS75 (i.e, 2054 for Afghanistan). We estimate t* based on projections of the demand satisfied indicator from FPEMcountry, with the maximum set to 2100. For countries with t* > 2030, the goal is to achieve the target in the target number of years (2030 – t ^(current)^), as opposed to in the BAU projected number of years (t* – t ^(current)^). We quantify the relative acceleration required to meet the DS75 target, *rel.accel _c_*, by comparing the number of years it would take to meet DS75 under the BAU scenario to the number of years until the desired year for target achievement (i.e., 2030):

$$rel.accel_{c}=({\text{t}}^{*}-{\text{t}}^{\left(\text{current}\right)})/(2030-{\text{t}}^{\left(\text{current}\right)}).$$

The relative acceleration measurement quantifies the speed up that would make this possible. For example, a required relative acceleration of 2 means that a country must achieve the target 2 times faster (or in half the number of years) than it is currently expected to do.

For countries with t* > 2030, the target-associated mCPR *tmod _c_* for 2030 is the mCPR projected for year t*:

$$t{\text{mod}}_{c}={\text{mod}}_{c,t*},$$

where mod _c,t*_ denotes mCPR in country c in year t* and *tmod _c_* is the DS75-associated target mCPR in country c. The difference between the levels of mCPR in *t ^(current)^* and the target mCPR is the current mCPR target gap, *gap _c_* given by:

$$gap_{c}=t{\text{mod}}_{c,}-{\text{mod}}_{c,t^{\left(current\right)}}.$$

Continuing with the Afghanistan illustration, Afghanistan needs to increase mCPR by 30.8% percentage points over an 11-year timescale in order to meet DS75 by 2030 rather than over the 35-year timescale that is currently projected for achieving DS75 (see Table 1).

**Table 1.** 75% demand satisfied (DS75) assessment. Countries are ordered by the target year in which they are projected to achieve DS75 under ‘business as usual’ (BAU) projections. *Modern contraceptive prevalence rate (mCPR) target gap* refers to the levels of mCPR required for DS75 to be achieved and *Users target gap* refers to the numbers of users that are required if DS75 is to be achieved by 2030.

Country	Target year	Target mCPR	mCPR target Gap (% point)	Users Target Gap (million)	Acceleration	DB mCPR gap
Sri Lanka	2031	58.7	4.3	0.02	1.1	58.9
Ethiopia	2032	53	15.3	6.35	1.2	52.1
Madagascar	2032	54.4	12.7	1.27	1.2	54
India	2036	57.2	7.3	27.74	1.5	56.7
Nepal	2040	59.3	12.9	1.44	1.9	58.8
United Republic of Tanzania	2040	55.5	18.1	3	1.9	52.9
Uganda	2041	58.2	20.8	2.6	2	55.8
Mozambique	2041	51.4	24.1	1.9	2	46.4
Djibouti	2047	53.5	28.6	0.05	2.5	46.9
Kyrgyzstan	2047	51.1	12.9	0.18	2.5	47.8
Burkina Faso	2047	51.4	21.6	1.42	2.5	46.2
Burundi	2048	55.3	28.5	0.72	2.6	50.8
Cambodia	2048	59.9	15.6	0.71	2.6	57.8
Senegal	2049	51.4	24.1	1.08	2.7	44.4
Sao Tome and Principe	2051	57.1	15.8	0.01	2.9	55.7
Liberia	2052	53.7	26.2	0.27	3	47.3
Timor-Leste	2053	55.8	29.1	0.08	3.1	50.6
Sierra Leone	2053	51.6	29.7	0.5	3.1	42.3
Bolivia (Plurinational State of)	2053	63.5	17.6	0.45	3.1	62.8
Afghanistan	2054	54.6	30.8	3.38	3.2	45.5
Niger	2054	48.1	29.1	2.12	3.2	37
Ghana	2055	55.4	26.7	1.45	3.3	49.5
Guinea-Bissau	2056	48.2	30.1	0.09	3.4	36.9
C√¥te d'Ivoire	2058	52.7	32.5	1.74	3.5	43.4
Congo	2060	55.3	29.8	0.33	3.7	50.7
Mali	2061	50.1	33.2	1.8	3.8	37.3
Comoros	2063	56.4	36.1	0.07	4	47.4
Pakistan	2064	56.5	29.7	15	4.1	47.5
Cameroon	2065	53.5	33	1.71	4.2	44.3
Philippines	2066	59.8	18.5	4.42	4.3	58
State of Palestine	2067	59.3	13	0.24	4.4	56.7
Mauritania	2067	52.5	37.7	0.35	4.4	40.1
Nigeria	2068	49.9	36.8	15.88	4.5	33.3
Guinea	2068	50.1	39.1	1.09	4.5	31.6
Eritrea	2069	53.5	41.2	0.27	4.5	38.3
Haiti	2070	59.6	26.3	0.51	4.6	57.1
Togo	2070	55	33.9	0.58	4.6	45.6
Tajikistan	2071	53.6	25.2	0.57	4.7	47.3
Benin	2074	52.8	39.6	1.08	5	40.1
Central African Republic	2076	51.2	36.1	0.41	5.2	38.6
Iraq	2076	59.3	20.4	2.46	5.2	54.7
Yemen	2076	60.6	26.4	1.97	5.2	55.1
Sudan	2077	52.5	38.6	3.42	5.3	37.1
Gambia	2086	51.6	41.3	0.21	6.1	31.9
South Sudan	2089	54.4	49.4	1.1	6.4	30.7
Democratic Republic of the Congo	2091	54.7	44.1	7.7	6.5	42.1
Somalia	2092	54.3	50.8	1.37	6.6	29.9
Solomon Islands	2100	48.4	22.3	0.03	7.4	43.7
Papua New Guinea	2100	50.4	21.8	0.45	7.4	46.1
Chad	2100	41	36.1	1.3	7.4	24.7

We compare the assessment of the AT-method-based mCPR gap associated with DS75 to the gap obtained by the approach used in New *et al.*, 2016 ^8^, referred to as the demand-based (DB) method. Under the DB approach, the target-mCPR is estimated as being 75% of the projected total demand in 2030. For Afghanistan, the DB approach gives a target mCPR of 45.3% (see Figure 1 and Table 1). This is lower than the AT method-based target mCPR of 54.6% because the DB approach is based on accelerated increases in mCPR only, as opposed to accelerated increases in both mCPR as well as total demand.

## Results

**Figure 2.**
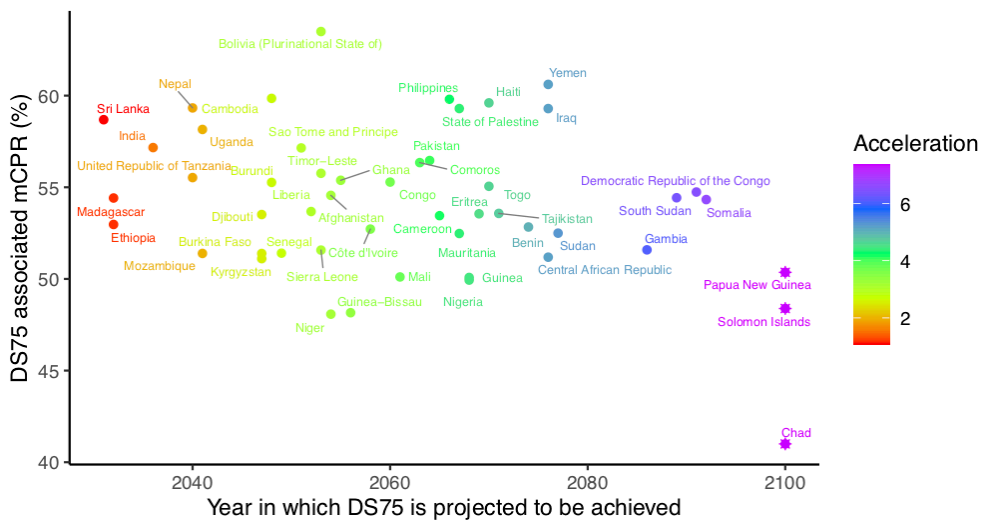
Target modern contraceptive prevalence rate (mCPR) plotted against 75% demand satisfied (DS75) target year. Colors indicate the relative acceleration required. Stars indicate countries where the target year is capped at 2100.

We constructed BAU projections for 68 countries of the FP2020 initiative and assessed the accelerations needed in each country to meet DS75 by 2030. In total, 50 out of 68 countries are projected to reach 75% demand satisfied in some year after 2030 and require acceleration in their FP transition between 2019 and 2030 to meet DS75 by 2030. Required relative accelerations range from 1.1 to greater than 7 ( Figure 2), with 35 countries needing to accelerate by at least a factor of 3 ( Table 1). Associated mCPRs among countries projected to meet DS75 before 2100 range from 48.1% in Niger to 63.5% in Bolivia (Plurinational State of). It is projected that DS75 will not be met by 2100 in three countries (Chad, Papua New Guinea and Solomon Islands). In these countries, the target mCPR was set to the projected mCPR for 2100, resulting in a relatively low target for Chad (41.0%).

For countries needing acceleration, we estimate that the mCPR target gaps between 2019 and 2030 range from 4.3 percentage points in Sri Lanka to 50.8 percentage points in Somalia ( Figure 2 and Table 1). mCPR gaps translate directly into the absolute numbers of required additional users of modern contraceptive methods. Nigeria and Pakistan face some of the biggest challenges needing to accelerate their modern contraceptive use transition by a factor of 4 in order to add 15.9 and 15.0 million additional users respectively by 2030 ( Table 1).

**Figure 3.**
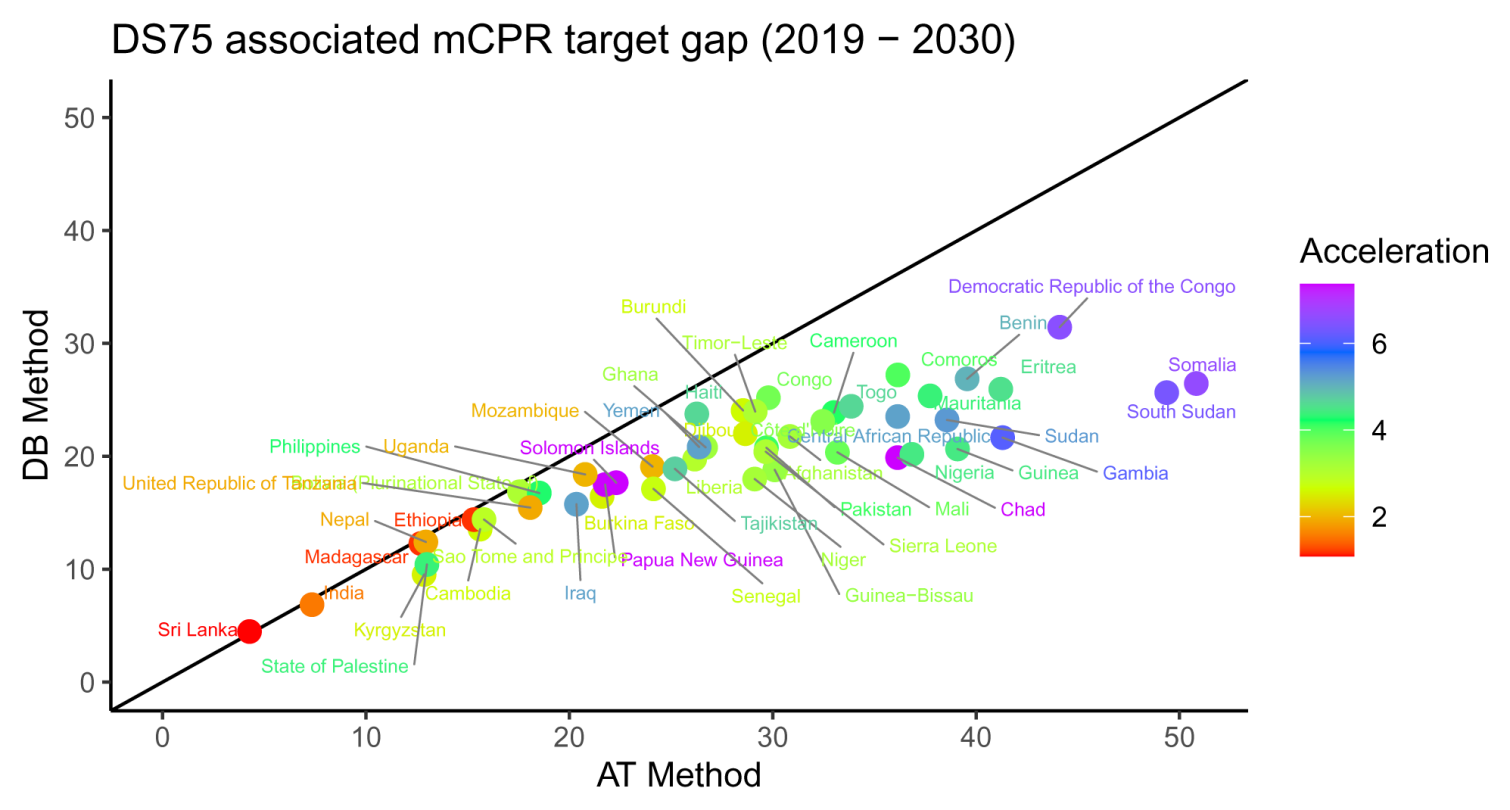
A comparison of the modern contraceptive prevalence rate (mCPR) target gaps according to the accelerated transition (AT) method and the demand based (DB) method. Each point represents a single country. Color indicates the relative acceleration needed according to the AT method. DS75 - 75% demand satisfied.

Figure 3 compares the estimated target gaps using the AT method to those obtained using the DB method. As expected, due to keeping demand in 2030 constant in the DB method, we observe that estimated target gaps are larger based on the AT method as compared to the DB method. The differences tend to be larger for countries with larger gaps that need more acceleration.

## Discussion

We have presented the accelerated transition (AT) method as a new method to calculate mCPR targets associated with DS targets. As the name suggests, the AT method quantifies the acceleration needed – as compared to business as usual projections – for a country to meet a target. We find that substantial accelerations are needed in countries that are not on track to achieve 75% demand satisfied for married or in-union women by 2030, with required mCPR increases ranging from 4.3 to 50.8 percentage points.

We suggest that the AT method provides more appropriate expectations for the levels of mCPR required to meet DS targets than the DB method ^8^. In countries where DS targets are projected to be later than 2030, we argue that the DB method fails to capture the dynamics of how FP indicators evolve. Specifically, the assumption of a fixed level of demand in 2030 accompanying the accelerated mCPR growth required to reach the target in this year is not realistic. That is, we cannot justify the assumption that accelerating mCPR will result in an equivalent deceleration of unmet need plus traditional contraceptive use combined. In fact, evidence suggests that increases in mCPR are likely to coincide with increased demand ^9^. To combat this, the AT method uses the FPEMcountry R package to estimate the mCPR in the year in which the DS targets in question is projected to be met, which implicitly accounts for the changes in demand that coincide with reaching this relevant level of demand satisfied. The comparison of the AT and DB methods for assessing DS75 illustrates that for countries with demand satisfied projected to be less than 75% in 2030 mCPR targets from the DB method are lower than AT-based estimates.

Recently, Li *et al.* also obtained mCPR targets for DS75 ^13^. They used a regression model to estimate mCPR as a quadratic function of DS, with country fixed effects. mCPR target values at DS75 were obtained by plugging in a DS of 75% in the fitted regression equation. Hence, the Li *et al.* mCPR targets are based on the assumption that differences in mCPR between countries stay constant with time and DS. While the model provides a good in-sample fit, its predictive performance is not verified ^13^. The approach also does not provide any measure of acceleration needed to accomplish DS75. In contrast, the AT method is based on the FPEM, which does not assume that differences in mCPR between countries stay constant with time and DS and has been shown to work well for short-term projection ^5, 6^. In addition, the AT method provides a measure of relative acceleration needed for the DS75 target.

While we suggest that the AT method improves upon existing methods, it is not without limitations. The AT method relies on projections of demand and mCPR by FPEMcountry. While this model has been shown to work well for short-term projections ^5, 6^, its accuracy for long-term projections cannot be verified. In addition, by definition, the method assumes that accelerated mCPR growth would promote the same changes in demand and unmet need as compared to seeing the same mCPR growth over a longer time period.

We demonstrated the use of the AT method here for evaluating the DS75 target. More general, the approach can be used for any DS-based target. Indeed, given the rapid acceleration needed for a large number of countries to achieve DS75 (35 countries need to accelerate their transition by at least a factor of 3), other country-specific approaches to target setting, i.e. based on attainment probabilities ^4^, may result in more realistic targets.

The main contribution of the AT method is to make DS-based targets actionable, that is, to provide the mCPR-associated target that in turn, provides direct information on the number of women needing modern contraceptives under the target. Given that accelerated progress towards demand-satisfied targets is desirable for many countries, we hope that the concrete information on the remaining gap in mCPR and associated users aids the implementation of successful FP programs.

## Data availability

### Source data

The processed survey data that support the findings of this study are openly available at https://www.un.org/en/development/desa/population/publications/dataset/contraception/wcu2019.asp and contained in the R package FPEMcountry ^7, 14^.

## Software availability

Source code is available from Github: https://github.com/FPRgroup/FPEMcountry/tree/v1.01

Archived source code at time of publication: http://doi.org/10.5281/zenodo.3899454 ^14^

License: MIT
