# Supplementary material for: What increase in modern contraceptive use is needed in FP2020 countries to reach 75% demand satisfied by 2030? An assessment using the Accelerated Transition Method and Family Planning Estimation Model
Source: Gates Open Res. 2020 Jul 21;4:113. [Version 1] doi: 10.12688/gatesopenres.13125.1 (PMC7578409; doi:10.12688/gatesopenres.13125.1)
Supplement: Peer review history

# Peer review: Reviewer response for version 1

**DOI:** 10.21956/gatesopenres.14307.r29381

**Published:** 2020-10-20

**Authors:** Amy O. Tsui

**Affiliations:** 1 Department of Population, Family and Reproductive Health, Johns Hopkins Bloomberg School of Public Health, Baltimore, MD, USA

This review is not written independently of the comments of the first Reviewer, many of which I agree with. 

 Evaluating and appreciating the article requires some background on global family planning, in particular the FP2020 initiative, the composite definition of demand satisfied (DS) and sources of data, as well as a working knowledge of Bayesian modeling. The article’s main contribution, besides its application of advanced statistical modeling, is, as it claims, to provide “actionable” DS targets.

 The subsample of countries for this analysis is the 50 of the 68 poorest countries in the world (as identified in 2012) which were the intended beneficiaries of the FP2020 initiative.

Comments (bold-face indicates edits/modification requested):

**The authors should clarify if the denominator of the contraceptive demand/need and use indicators is all women of reproductive age or women in union only, or both.  If both, clarify how including only married women will bias the estimates, particularly in sub-Saharan African countries. Are the denominators for these rates consistently defined for each country?**

 Demand Satisfied (DS) for women of reproductive age is not defined in equation form and is:

             DS = mCPR/(CPR + UN)

 where mCPR is the Modern Contraceptive Prevalence Rate and CPR is the Contraceptive Prevalence Rate, irrespective of method type. Unmet Need is the percent of women of reproductive age who desire to postpone or end childbearing but are not currently using a contraceptive method; it is also a “prevalence rate” (which enables summing it with CPR to represent Total Demand). 

 The Accelerated Transition (AT) method described in this article involves setting the value of mCPR for each country for 2030 (when the Sustainable Goal Target 3.7 is to be achieved) to be the same value in whatever year the Family Planning Estimation Model projects 75% of DS will be reached. Its limitation is that although mCPR is artificially accelerated, what happens to the denominator components of CPR and UN under acceleration is not known. Presumably CPR will increase proportionally (without, for example, a large increase in traditional method use) and UN will decline linearly. However, research has shown that UN can increase at first with a CPR increase before it decreases (e.g., Westoff, 2001). This could offset the 75% satisfied level. **Please comment on whether you expect the DS75 level in 2030 should match what is suggested by FPEM should the mCPR indeed be significantly accelerated, or if it is not important.**

 The target mCPRs (see Table 1) for DS75 are nearly all around 50% and the average number of years of acceleration is 31.7 (target years minus 2030). Thus many of these countries are unlikely to reach their target mCPR in 2030. **Please comment on how realistic these projections are given their intended actionability. How are these quantified targets likely to be a more effective call to action than FP2020 was? **Note that the additional users in 2019 per the FP2020 Data Dashboard for FP2020 countries is 53.4 million, or a gap of 66.6 million against the 120 million goal.  Five countries in Table 1 appear to account for a user gap of 62.1 million (Ethiopia, India, Philippines, Nigeria, and the DR Congo). **Please comment on whether a call to action should focus on accelerating satisfying demand in primarily populous or all 50 countries despite the low likelihood of achieving the target in many.**

 The influence of contraceptive method mix is not discussed. India’s mix profile is heavily dominated by contraceptive sterilization which would indicate a larger demand for limiting than spacing childbearing. Contrast this with SSA countries with more traditional method use (e.g., Central African Republic) or a high percentage of injectable method use, where demand to space births is greater. **Please comment on how shifts in contraceptive method mix either due to new supply developments or age-related use will affect the target mCPRs and “users target gaps”.**

Minor comments:

- Ensure the use of the acronym BAU (Business as Usual) is consistent throughout the paper; sometimes it is BUA and sometimes BAU. As Reviewer 1 requests, clarification of the BAU description is warranted. As I understand it, BAU is simply assuming current trend for a given country follows the projection from the Family Planning Estimation Model. (It’s not clear to me why relabeling this method as BAU is needed.) 
- The FPEM is used on a country-specific basis for the 50 FP2020 countries. This is referred to as the one-country implementation of FPEM. Why not just say it’s a country-specific application of FPEM? One-country is an awkward term.
- Since both the AT method and the regression method in Li e *t al.* (2019) (referenced in the article) have a similar aim to provide target mCPRs for 75% demand satisfied levels but their predictive performances are not yet verified, it will be interesting to compare actual mCPRs in 2030 with those estimated by these two methods. **It is worth briefly commenting in the Discussion section what the role of behavioral factors is in driving contraceptive use, as this seems to be an absent consideration for statistical modeling/assessments.**

Is the rationale for developing the new method (or application) clearly explained?

Yes

Is the description of the method technically sound?

Yes

Are the conclusions about the method and its performance adequately supported by the findings presented in the article?

Yes

If any results are presented, are all the source data underlying the results available to ensure full reproducibility?

Partly

Are sufficient details provided to allow replication of the method development and its use by others?

Yes

Reviewer Expertise:

contraceptive behavior, reproductive health, demography

I confirm that I have read this submission and believe that I have an appropriate level of expertise to confirm that it is of an acceptable scientific standard.

# Peer review: Reviewer response for version 1

**DOI:** 10.21956/gatesopenres.14307.r29500

**Published:** 2020-09-21

**Authors:** Shawn Malarcher

**Affiliations:** 1 Office of Population and Reproductive Health, United States Agency for International Development (USAID), Washington, D.C., USA

The article describes a new method for estimating how much additional effort will be needed to achieve goals in demand satisfied for family planning by 2030. These estimates will provide country leaders and decision-makers much needed information to assess if their goals are achievable and how much additional effort may be required to meet those goals. The authors provide comparison with previously used methods which does not adjust unmet need for changes over time. The authors provide sufficient justification that the revised method is an improvement over the current standard.

 As my focus was mainly related to use and interpretation of the analysis and results, I highly recommend ensuring review by someone with expertise in modeling to review methods used. I hope the authors will consider the following to further strengthen this important piece of work:

  

- This statement is circular, please clarify/correct. “Estimates of unmet need are obtained by capturing the relationship between contraceptive prevalence and unmet need.”
- In general, I found the description of BAU unclear. Consider reworking with more focus on plain language and contrasting the difference between BAU and the accelerator.
- The article would benefit from a stronger framing of the analysis in expanding access and choice. For example, the following statement “Countries that are not currently projected to meet DS75 by 2030 will require a larger-than-BUA increase in the number of users of modern methods of contraception between now and 2030.” could be restated as, “Countries that are not currently projected to meet DS75 by 2030 will require an examination of factors contributing to low contraceptive use and restricted access to family planning services, in these context the “BUA” approach is not addressing the values and preferences of large proportions of the population with an unmet need.”
- The point of this scenario is unclear, “A previous study for states in India has estimated the required increases in mCPR and additional users needed to achieve DS targets using 2030 projections of total demand (contraceptive prevalence plus unmet need) for contraception ^8^ . This method combined accelerated growth in modern use with business-as-usual growth in total demand; the method did not account for faster-than-BAU increases in mCPR to coincide with faster-than-BAU increases in demand that are likely to occur due to, for example, increased efforts in family planning programs ^9^. Methods that account for faster-than-BAU increases in demand to coincide with faster-than-BAU increases in mCPR are needed to avoid underestimation of mCPR targets, and thus, failure to meet DS targets.” What did the estimation show? How did it influence India’s investments?
- Please clarify: “Afghanistan needs to increase mCPR by 30.8% percentage points over an 11-year”. Is this 30.8 percent of the percentage point increase or an increase of 30.8 percentage points?
- Please clarify: “Associated mCPRs among countries projected to meet DS75 before 2100 range from 48.1% in Niger to 63.5% in Bolivia (Plurinational State of).”
- Please clarify if the model uses mCPR vs CPR in the estimation of unmet need. Both measures are referenced in the article.
- Please include a discussion of the age of data in the limitation section. How many of these countries have data that is more than 5 or even 10 years old? How accurate is that data?
- Please expand your description of the “acceleration factor”. How should this be understood? What does it mean for a country to have an acceleration factor or 4 versus 2?
- Please provide some discussion of how COVID 19 effects this analysis.

Is the rationale for developing the new method (or application) clearly explained?

Yes

Is the description of the method technically sound?

Partly

Are the conclusions about the method and its performance adequately supported by the findings presented in the article?

Yes

If any results are presented, are all the source data underlying the results available to ensure full reproducibility?

Yes

Are sufficient details provided to allow replication of the method development and its use by others?

Partly

Reviewer Expertise:

Research Utilization. Population and Reproductive Health

I confirm that I have read this submission and believe that I have an appropriate level of expertise to confirm that it is of an acceptable scientific standard.
